# Does Eyewitness Confidence Calibration Vary by Target Race?

**DOI:** 10.3390/bs16020257

**Published:** 2026-02-10

**Authors:** Dilhan Töredi, Jamal K. Mansour, Sian E. Jones, Faye Skelton, Alex McIntyre

**Affiliations:** 1 Department of Psychology, Åbo Akademi University, Tuomiokirkontori, 320500 Turku, Finland; 2 Division of Psychology, Sociology and Education, Queen Margaret University, Drive, Musselburgh EH21 6UU, UK; jamal.mansour@uleth.ca (J.K.M.); sjones@qmu.ac.uk (S.E.J.); 3 Department of Psychology, University of Lethbridge, 4401 University Dr W, Lethbridge, AB T1K 3M4, Canada; 4 School of Applied Sciences, Edinburgh Napier University, 9 Sighthill Ct, Edinburgh EH11 4BN, UK; f.skelton@napier.ac.uk (F.S.); a.mcintyre@napier.ac.uk (A.M.)

**Keywords:** cross-race effect, accuracy, CAC, confidence, calibration

## Abstract

After making a lineup decision, eyewitnesses may be asked to indicate their confidence in their decision. Eyewitness confidence is considered an important reflector of accuracy. Previous studies have considered the confidence-accuracy (CA) relationship—that is, the relationship between participants’ confidence in their lineup decision and the accuracy of that decision. However, the literature is limited and mixed concerning the CA relationship in cross-race scenarios. We considered the CA relationship for White and Asian participants and targets (fully crossed) using sequential lineups. Participants completed four trials (two White targets and two Asian targets). For each trial, they watched a mock-crime video, performed a distractor task, made a sequential lineup decision (target-present or target-absent), and indicated confidence in their lineup decision. White participants had higher identification accuracy with White than Asian targets, while Asian participants were similarly accurate with White and Asian targets. White participants’ confidence was better calibrated for White than Asian targets, except for when they had medium-high confidence (no difference). This finding is not only theoretically relevant—showing support for the optimality hypothesis—but also practically relevant—suggesting that the CA relationship may differ for target races at some levels of confidence.

## 1. Does Eyewitness Confidence Calibration Vary by Target Race?

After making a lineup decision, eyewitnesses may be asked to indicate their confidence in their decision. Eyewitness confidence is considered an important indicator of accuracy (Wixted & Wells, 2017). For instance, the U.S. Supreme Court has approved confidence as one of five factors to consider when evaluating identification accuracy (Neil v. Biggers, 1972). Likewise, law enforcement, prosecutors, defence attorneys, and potential jurors all share the belief that confidence is a crucial indicator of an eyewitness’s accuracy (Deffenbacher & Loftus, 1982; Noon & Hollin, 1987; Potter & Brewer, 1999). Additionally, studies of mock jurors have revealed that confidence substantially impacts their assessments of witness credibility and verdicts (Cutler et al., 1988; Garrett et al., 2020; Slane & Dodson, 2022; Vallano et al., 2019). For example, Cutler, Penrod, and Stuve found that, of ten variables known to affect eyewitness identification accuracy, only eyewitness confidence consistently influenced mock jurors’ perceptions of eyewitness accuracy and defendant guilt.

Eyewitness confidence can be a reliable reflector variable—a variable that elicits responses which can be used to differentiate guilty from innocent suspects (Wells, 2020)—when the eyewitness’s memory has been protected from contamination (Wixted & Wells, 2017; see Sauer et al., 2019 for discussion of boundary conditions). Eyewitness confidence has been shown to be a stronger predictor of lineup performance than other indices of reliability, such as how long an eyewitness takes for an identification (Carlson et al., 2025), underscoring the need for particular attention to confidence as a reflector variable. Previous studies have considered the confidence–accuracy (CA) relationship—that is, the relationship between participants’ confidence in their face recognition decision (e.g., old/new paradigm, lineup paradigm) and the accuracy of that decision. A strong CA relationship means that high confidence is reliably associated with a high probability of accuracy, while low confidence is reliably associated with a low probability of accuracy. However, the literature is limited and mixed concerning the CA relationship in cross-race scenarios, as we will discuss below.

It is important to consider cross-race scenarios because individuals recognise faces that belong to their own race better than faces of another race, a phenomenon referred to as the cross-race effect (i.e., CRE; Lee & Penrod, 2022; Meissner & Brigham, 2001). Given that cross-race identifications, like same-race identifications, are used as evidence in court, an understanding of how eyewitnesses make confidence judgments when they make cross-race identifications is of value to the criminal justice system. Confidence is often treated as a reliable indicator of accuracy by legal decision-makers, yet the mechanisms underlying confidence judgments may differ as a function of target race. A real-world illustration of this issue is the case of Ronald Cotton and Jennifer Thompson-Cannino. Cotton, a Black man, was wrongfully convicted largely based on a White eyewitness’s highly confident—but mistaken—identification. This case, documented by the National Registry of Exonerations (2024), highlights the potential consequences of relying on eyewitness confidence in cross-race identification contexts without a nuanced understanding of its diagnostic value.

Three studies have reported a positive CA relationship regardless of target race (Brigham et al., 1982; Josephson & Holmes, 2011; Nguyen et al., 2017). For instance, Josephson and Holmes found that confidence predicted accuracy across White and Black participants using White and Black faces, although their sample was small (only 10 participants per group). However, other studies have found that participants give higher confidence ratings for correct recognition responses and lower confidence ratings for incorrect recognition responses when same-race faces are considered compared to when cross-race faces are considered (e.g., Dodson & Dobolyi, 2016; Grabman et al., 2019; Horry & Wright, 2008; Rhodes, 2013). In this study, we aimed to investigate the CA relationship with White and Asian participants and targets (a fully crossed design; Lee & Penrod, 2022) using a lineup paradigm. The only other study that used a lineup paradigm and a fully crossed design with Asian and White participants found that confidence predicted identification accuracy for same-race choosers, but not cross-race choosers (S. M. Smith et al., 2001). This was the case regardless of the participant’s race. The mixed and limited findings on the confidence-accuracy relationship in cross-race situations highlight the importance of further examining this topic. Notably, most cross-race research has focused on White and Black faces, and little research has demonstrated the generalisability of the CRE across other race pairings (Töredi et al., 2024).

Moreover, only one published study known to the authors adopted a sequential lineup paradigm—showing lineup members one at a time—to study the CA relationship with same-race and cross-race targets (Wylie et al., 2015). It is important to consider the CA relationship with sequential lineups as they are widely used in several countries, such as the UK, Canada, and parts of the US (Fitzgerald et al., 2021). Though Wylie and colleagues used sequential lineups, they calculated correlations between correct decisions and confidence to investigate the CA relationship. Correlations are considered an outdated method to test the CA relationship (Brewer & Wells, 2006; Olsson & Juslin, 2002). A correlation coefficient (e.g., Pearson’s *r*) provides a single summary statistic that describes the strength and direction of the linear relationship between confidence and accuracy. However, it does not capture the nuances of the CA relationship, such as how suspect identification accuracy differs at different levels of confidence. For this reason, we used more rigorous indices of the CA relationship and investigated this relationship using sequential lineups.

In the current study, we used regression models, CAC curves (Mickes, 2015), and calibration indices (Brewer & Wells, 2006) to investigate the CA relationship. Regression models show whether confidence predicts accuracy. When target race is included in regression models, the model can show how much confidence changes as accuracy changes, as well as the extent to which this differs when target race varies. CAC curves (Mickes, 2015) illustrate suspect identification accuracy at discrete levels of confidence (e.g., when the eyewitness is between 90% and 100% confident). Separate CAC curves for each target race and participant race can provide a clear visualisation of the CA relationship in each circumstance. Finally, three widely used calibration indices are the calibration index (c), over/underconfidence (O/U), and the adjusted normalised discrimination index (ANDI; Yaniv et al., 1991). The calibration index indicates how well confidence aligns with accuracy across levels of confidence. Over/underconfidence indicates the extent to which eyewitnesses are more or less confident than their accuracy warrants. ANDI indicates how well confidence differentiates between accurate and inaccurate eyewitness identifications. Several studies have used these measures (e.g., Lindsay et al., 2013; Weber & Brewer, 2004); however, they have only rarely been considered in a cross-race scenario.

Dodson and Dobolyi (2016) conducted maybe the most comprehensive examination to date in consideration of the CA relationship given different races, as they reported a variety of analyses (i.e., calibration curves, regressions, calibration indices, though not CAC curves) for a study with White and Black participants and targets. When participants (regardless of race) chose a face from a lineup, their confidence was more closely linked to accuracy when choosing a same-race face compared to a cross-race face. The authors argued that the CA relationship differences based on target race occurred because participants were more overconfident when choosing cross-race faces at lower levels of confidence than when choosing same-race faces, even though they were overconfident in both cases. Notably, the differences in effect sizes between the groups were relatively small. Furthermore, Grabman et al. (2019) found that when eyewitnesses were highly confident (cf. medium- or low-confidence), the CRE was smaller. More recent work has replicated these findings. Helm and Spearing (2025; Study 1) showed that participants were better calibrated for same-race targets and more overconfident for cross-race targets across all confidence levels. Taken together, these results suggest that confidence calibration is superior in same-race lineups, whereas cross-race lineups tend to elicit overconfidence.

## 2. Current Study

Given that the literature examining the CA relationship across race pairings is often limited in scope and largely focused on White–Black race pairings using simultaneous lineup procedures, the present study extends the literature by examining the CA relationship in White–East Asian race pairings using sequential lineups and up-to-date analytic methods. This extension is important because both lineup procedure and target race can systematically influence eyewitness decision-making (see Wylie et al., 2015). Moreover, given the substantial weight jurors place on eyewitness confidence (Garrett et al., 2020), it is critical to evaluate the CA relationship beyond the commonly studied racial pairings and lineup formats. By addressing this gap, the current research enhances the generalizability of CA findings and provides evidence that reflects the diversity of eyewitness identification contexts encountered in real-world investigations.

Aligning with previous findings, we expected to find a cross-race effect, and we expected confidence to moderate the size of the CRE. We expected that confidence would be predictive of identification accuracy (i.e., a valid reflector variable; Wells, 2020), but that confidence would be a better predictor of same-race than cross-race identification accuracy. We also explored whether previous findings (e.g., Dodson & Dobolyi, 2016; Helm & Spearing, 2025) of greater overconfidence with cross-race compared to same-race faces would replicate.

## 3. Method

### 3.1. Participants

The final sample had participants self-identifying as Asian (*N* = 203) and White (*N* = 202). Participants were recruited via Mechanical Turk/CloudResearch (MT/CR)—received monetary compensation of $2.50—and university participant pools—received course credit. Due to limited resources, all Asian participants were recruited via MT/CR, whereas most White participants were recruited via university participant pools. As part of the broader doctoral thesis from which this study is drawn (see Töredi, 2024), the same lineups were examined across two recruitment sources (MTurk/CloudResearch and university samples), with results indicating minimal differences in lineup fairness and performance.

#### 3.1.1. Sample Size Determination

The required sample size was estimated to be 200 participants per participant race to have 80% power to detect the cross-race effect. This was estimated using a simulation conducted in R Version 2024.09.1+394 (R Core Team, 2022) that assumed an odds ratio of 1.4 for correct identifications (Meissner & Brigham, 2001), considered sample sizes from 150 to 1000, in 50 participant increments, and reported how often a significant CRE (i.e., *p* < 0.05) would occur in a multilevel logistic regression with target race (same-race, cross-race) as a predictor of correct identifications and estimating a random effect for participant.

#### 3.1.2. Data Inclusion and Exclusion

Individuals could participate in this online study provided they were 18 or more years old, using a laptop or a desktop computer, and spoke and understood English. For data collection from the university participant pools, 17-year-old participants were allowed in order to ensure inclusivity per the National policy of persons 16 or older being considered adults for such a purpose. Participants were required to complete the study on a laptop or desktop computer in order to ensure they had a clear enough view of the targets to allow them to identify them from a lineup. Only participants who self-identified as Asian or White were recruited via MT/CR; participants of any ethnicity were recruited via university participant pools. Participants who did not self-identify as Asian or White were excluded from the final analysis. Participants could quit the study at any time and, except for demographic questions, were required to answer all questions. Only participants who responded to all experimental questions and passed at least one of three attention-check questions were included in the analyses. After reading the debriefing, participants were given the option to withdraw or submit their data. For those who chose to withdraw, their data were not included in the analyses. Finally, participants who reported technical issues that affected their view of the mock-crime video or lineups (e.g., the video/image did not appear) or reported having cheated in a way that would influence their results (e.g., by taking a picture of the target) were excluded from analyses.

Initially, 1012 individuals were recruited, but only 461 consented and completed the survey in full. Fifty-six participants were excluded from the analyses: nine did not consent for their data to be included, 14 failed all three attention checks, and 17 had technical problems. Six participants self-identifying as Black, two participants self-identifying as Hispanic/Latin, and six participants who preferred not to answer the race question were excluded. One participant was excluded because they stated that they took a picture of the target, and another participant was excluded because they self-reported that they were a “beginner” with English.

#### 3.1.3. Demographics

Asian participants ranged in age from 18 to 69 years (*M* = 34, *SD* = 9.69; four preferred not to answer). Participants self-identified as male (*n* = 100), female (*n* = 99), non-binary/third gender (*n* = 2), or preferred not to answer (*n* = 2). Participants were mostly native English speakers (*n* = 147), followed by fluent (*n* = 52), advanced (*n* = 3), or intermediate (*n* = 1). Furthermore, 110 of the Asian participants reported that they were originally from the USA, while 62 reported being from a range of other countries^1^ (32 preferred not to answer). Similarly, 187 responded that they currently resided in the USA, with two in Pakistan and one in Kazakhstan (13 preferred not to answer). Finally, 149 reported living longest in the USA, and 34 in other countries^2^ (21 preferred not to answer).

White participants ranged in age from 17 to 78 (*M* = 24.02, *SD* = 10.58; three participants preferred not to answer). Thirty-seven identified as male, 162 as female, and three as non-binary/third gender. Most participants had a native level of English (*n* = 165), 35 were fluent, and two reported they were at an advanced level. Furthermore, 141 of the participants reported that they were originally from the UK, 31 from the USA, and 22 from a range of other countries^3^ (eight participants preferred not to answer). Similarly, 165 respondents responded that they were residents in the UK right now, 29 in the USA, and one in Egypt (eight participants preferred not to answer). Finally, 149 reported living longest in the UK, 28 in the USA, and 16 in other countries^4^ (seven participants preferred not to answer).

### 3.2. Design

The study was a 2 (Target Presence: target-present, target-absent; within-subjects) × 2 (Target Race: Asian, White; within-subjects) × 2 (Participant Race: Asian, White; between-subjects) × 2 (Target: a, b; within-subjects) mixed design. Target Presence refers to whether a lineup shown to participants contained the person shown in a watched mock-crime video. Participant Race refers to whether a participant self-identified as Asian or White. Target Race refers to whether a mock-crime video depicted an Asian or a White actor. Target refers to the fact that the mock-crime videos featured different actors (a, b) to ensure the findings were not unique to individual actors (Wells & Windschitl, 1999). The order of the video/lineup trials was randomised, and participants were randomly assigned to conditions.

### 3.3. Materials

#### 3.3.1. Mock-Crime Videos

Four mock-crime videos were used. Each showed an Asian or White male stealing money from a purse. All actors were in their early 20s, wearing their own clothes. These videos came from a set of videos and lineups developed for research, though the specific videos used vary (e.g., Beaudry et al., 2015; Mansour et al., 2009). Each video was approximately 30 s long. The display of the videos was clear and of good quality for viewing. Prior to seeing any videos, participants were informed that they would watch four videos with audio and answer questions about them.

#### 3.3.2. Intervening Task

Participants completed a 30 s intervening task between each mock-crime video and lineup to disrupt their ability to rehearse their memory for the target and to ensure subsequent lineup decisions relied on long-term memory rather than working memory, as would occur in the real world. The intervening task involved viewing a Where’s Waldo^5^ image that depicts a beach. Participants were instructed to answer as many questions as they could about this image during the allotted time. The questions were designed to be impossible to complete in the allotted time. For example, the questions included: “*How many open umbrellas are there?*” and “*Does the man in the red-and-white bathing suit have a moustache?*”.

#### 3.3.3. Lineups

Six-person target-present and target-absent photographic lineups were used. White lineup stimuli were from the same set as those used in Mansour et al. (2009), though the specific lineups used vary. Asian lineup stimuli were developed using the match-to-description technique (Navon, 1992; Luus & Wells, 1991; Koehnken et al., 1996). Lineups for White targets were constructed using the same method, but previous to our use of them. The descriptions used for all lineups were derived from modal descriptions of the targets previously obtained in the lab.

For the Asian lineups, ten volunteers (six female, four male) self-identifying as East Asian (as lineup constructor race can influence the lineup construction, see Brigham & Ready, 1985; Akan et al., 2025) were given the filler pool (i.e., all East Asian faces in the lab’s database of faces) and the descriptions. They sorted the faces into “matching description” and “not matching description” categories for each target. This process resulted in eight faces for one target and nine for the other. Volunteers were not instructed to choose a specific number of faces. Next, a second group of ten volunteers who self-identified as East Asian (three female, seven male) viewed each of the description-matched face sets and were asked to report any faces that stood out or did not look similar to the others in the set. Faces were excluded if at least half of the volunteers agreed the face stood out or was less similar to the other faces. This process resulted in exactly six faces for each target. These six faces were used as fillers in our lineups.

For each target, the target-present lineup contained the target and five fillers (chosen randomly out of six), while the target-absent lineup contained six fillers. No fillers were repeated across lineups. The positions of the target and fillers were randomised across participants and across lineups, such that each participant saw a different configuration for each lineup. This was to ensure our findings were not influenced by suspect positioning effects (e.g., Carlson et al., 2008). There were no designated innocent suspects. These lineups depicted the lineup members one by one, and participants viewed all lineup members twice (same positioning as the first time) before providing a decision (in line with how lineups are presented in the UK). Before each lineup, participants read unbiased lineup instructions that emphasised that the criminal may or may not be in the lineup (Brewer & Wells, 2006). Specifically, they were informed that:

The video you saw showed a mock-crime. We would now like to show you a lineup (an array of photographs of faces) for the “criminal” you saw in the video. Please view each member carefully. Please note that the “criminal” you saw may or may not be in the lineup. You should remember that it is just as important to clear innocent persons from suspicion as to identify the guilty. After you have seen the lineup, you will be asked for the number of the “criminal” or to indicate that they were not present. If you see the “criminal”, please make sure you keep the number of that photo in your mind.

The resultant Tredoux’s *E*s (A. M. Smith et al., 2021; Tredoux, 1998) for the White target-absent lineups were 3.48, 95% CI [2.44 to 6.02], and 4.11, 95% CI [3.26 to 5.56] based on White participants’ decisions. For the Asian target-absent lineups, the resultant Tredoux’s *E*s were 1.55, 95% CI [1.23 to 2.09] and 2.36, 95% CI [1.83 to 3.33] based on the Asian participants’ decisions. Higher values of Tredoux’s *E* (closer to nominal size) indicate greater fairness because more lineup members are considered good alternatives to choose from by eyewitnesses. Resultant Tredoux’s *E* reflects the choosing behaviour of eyewitnesses in the current experiment (cf. mock witnesses) and is a better predictor of lineup fairness than mock witness measures (Lee et al., 2024).

### 3.4. Measures

#### 3.4.1. Lineup Responses and Eyewitness Confidence

We coded all lineup decisions as either correct identifications (suspect identifications from target-present lineups) or any other identification (filler identifications from target-present and target-absent lineups) to produce our measure of identification accuracy.

#### 3.4.2. Eyewitness Confidence

Following the lineup decision, participants were asked to indicate their confidence in their decision on a 0–100% (0 = *Not at all confident*, 100 = *Extremely confident*) scale.

#### 3.4.3. Demographics and Data Quality Checks

We asked the participants six demographic questions (age, sex, English level, country of origin, country of residence, and country they lived longest in), for which they had an option of not responding. We also asked three attention-check questions about the mock-crime videos (e.g., “*What was the colour of the purse from which the “criminal” stole the money?*”) to determine whether participants paid sufficient attention to the mock-crime video to be included in the analyses.

We asked four multiple-choice questions to assess data quality. We asked about whether participants experienced technical difficulties (*Did you experience any technical difficulties with viewing the images [*e.g., *could not hear or see one or both]?*) and whether participants cheated on the task in any way (*When doing this study*, *did you cheat in any way?*), whether participants had completed an eyewitness study before (*“Have you ever done a study on eyewitness memory before?”*) and whether participants had seen a similar mock-crime video before (*“Have you ever seen a mock-crime video like the one we showed you before?”*). For the technical difficulties and cheating questions, if participants responded yes, they were asked to expand on their answer so we could assess whether their data should be excluded. Answers to other questions in this paragraph were not used as exclusion criteria but rather to inform the lab about the naivety of our samples. For all questions described in this section, participants were assured that their responses would not influence whether they received credit/payment.

### 3.5. Procedure

Participants completed four lineup paradigm trials. On each trial, they watched a mock-crime video, worked on the Where’s Waldo task, made their lineup decision, and indicated their confidence in their lineup decision. Following this, participants answered the potential CRE measurement items (see Töredi et al., 2025), demographic questions, attention check questions, and data quality questions. Finally, participants were debriefed and reimbursed/credited as appropriate.

## 4. Results

All data coding and analyses were conducted in R (R Core Team, 2022). Beyond the base built-in R functions, analyses were conducted using the *Tidyverse repository* (Wickham et al., 2019) and functions within the following packages: *ggplot* (Wickham et al., 2019), *data.table* (Dowle & Srinivasan, 2023), *magrittr* (Bache & Wickham, 2020), *lme4* (Bates et al., 2015), *boot* (Canty & Ripley, 2022), *janitor* (Firke, 2023), and *sjmisc* (Lüdecke et al., 2021). For all analyses, *p*-values less than 0.050 were regarded as statistically significant.

### 4.1. Lineup Decisions

To test the CRE, multilevel binomial logistic regressions with participant as a random effect and Target Race as a fixed effect were employed to predict identification accuracy. Separate analyses were conducted for White and Asian participants. Visualisations of lineup decisions, resultant lineup fairness for target-present and target-absent lineups, and lineup member selections (see Tables S1–S3 in Supplementary Materials) showed that one of the Asian target-absent lineups was a biased lineup. Therefore, the Asian targets’ lineups were removed from subsequent analyses. Analyses using the full set of lineups (per our preregistration: https://osf.io/j9nzc/overview, accessed on 20 February 2021) are reported in the Supplementary Materials. For White participants, there was a CRE for identification accuracy, *z* = 2.21, *p* = 0.02. For Asian participants, there was no CRE for identification accuracy, *z* = 1.12, *p* = 0.26.

In order to estimate discriminability in a forensically relevant way, we also computed discriminability using target-absent filler identification rates (i.e., by dividing the total target-absent filler identification rate by the resultant Tredoux’s *E*; Quigley-McBride & Wells, 2021). White participants had a higher average discriminability for White targets (*d′* = 2.45) than for the Asian target (*d′* = 1.79). Similarly, Asian participants had higher discriminability for the White targets (*d′* = 2.16) than for the Asian target (*d′* = 1.98). To inferentially assess whether Target Race influenced discriminability (i.e., the ability to differentiate guilty from innocent suspects) and response criterion (i.e., the evidence threshold that determines whether an eyewitness identifies someone; DeCarlo, 1998; Wright & London, 2009), we used multilevel probit logistic regression models. The models predicted lineup decision accuracy (correct identifications and filler identifications from target-absent lineups coded as 1, and all other lineup decisions as 0) with Target Presence, Target Race, and their interaction as fixed effects and participant as a random effect. For White participants, the interaction was significant, indicating that discriminability was lower for Asian (*d′* = 0.88) than White targets (*d′* = 1.39), *z* = 1.97, *p* = 0.04. For Asian participants, discriminability was similar for Asian (*d′* = 1.10) and White targets (*d′* = 1.19), *z* = 0.30, *p* = 0.75.

### 4.2. Confidence-Accuracy Relationship

Confidence was expected to be a reflector of accuracy for White and Asian participants. To examine this, confidence was regressed to predict identification accuracy in a model that also included participant as a random effect. Confidence predicted identification accuracy for both White participants, *z* = 8.14, *p* < 0.001, and for Asian participants, *z* = 6.55, *p* < 0.001. Because Asian participants did not exhibit a CRE for identification accuracy, it is not informative to consider their CA relationship for White and Asian targets. Therefore, we only report the CA relationship findings of the White participants in subsequent sections; the results for Asian participants are reported in the Supplementary Materials.

We examined whether confidence interacted with the Target Race to predict lineup accuracy—again using a multilevel binomial logistic regression model, but now included Target Race, confidence, and their interaction as fixed effects, as well as participant as a random effect. It was hypothesised that confidence would interact with Target Race. The Target Race by confidence interaction was significant for White participants, *z* = 2.46, *p* = 0.01. This result is consistent with our hypothesis of a stronger CA relationship for same-race than cross-race targets. We next constructed CAC curves to determine if the nature of the interaction was as hypothesised.

Separate CAC curves (Mickes, 2015) were plotted for each Target Race. Since this study did not use a designated innocent suspect, the innocent suspect identification rate was estimated by dividing the target-absent filler identification rate by the lineup’s resultant functional size (Tredoux, 1998; A. M. Smith et al., 2020; Fitzgerald et al., 2023). Confidence was categorised as low (0–50%), medium-low (51–70%), medium-high (71–90%), or high (91–100%), and accuracy was plotted in each confidence bin. Identity lines were determined by calculating the weighted mean accuracy for each confidence bin. Good calibration occurs when a CAC curve closely follows the identity line (y ≈ x). When the curve lies above this line, it indicates underconfidence. Conversely, when the curve falls below the identity line, it indicates overconfidence.

**Figure 1:**
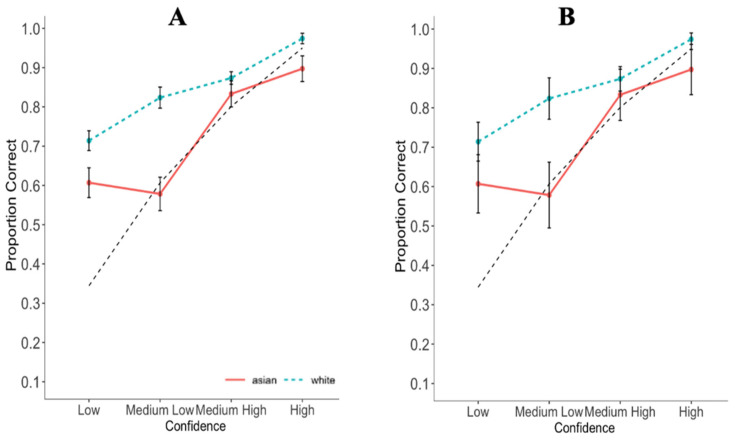
Confidence Accuracy Characteristic Curves by Target Race, using Standard Error Bars and 95% Confidence Intervals. Note. (**A**) includes standard error bars, while (**B**) includes 95% confidence intervals. Red straight lines represent Asian targets while teal dashed lines represent White targets.

The CAC curves for each Target indicated a positive CA relationship (see Figure 1). For low-confidence identifications and medium-high confidence identifications, White participants were underconfident with both White and Asian targets. Medium-low confidence identifications were well-calibrated for Asian targets but not for White targets, for which they were underconfident. Finally, participants were slightly underconfident with White targets and overconfident with Asian targets when they had high confidence.

Figure 1 displays the CAC curves separately with standard error bars (Panel A) and 95% confidence intervals (Panel B) in order to provide comprehensive insight into potential differences between confidence bins across Target Race conditions. Overlapping standard errors indicate a difference is not statistically significant. Standard errors that do not overlap are uninformative about statistical significance. Non-overlapping 95% confidence intervals indicate a statistically significant difference when α = 0.05. 95% confidence intervals that overlap are uninformative about statistical significance. Therefore, using both standard errors and confidence intervals allowed us to judge where statistically significant differences did and did not occur. Specifically, non-overlapping confidence intervals indicated a statistically significant difference between Target Race conditions. When confidence intervals overlapped (and were thus uninformative regarding significance), we examined standard errors for more information. If the standard errors overlapped, this indicated a failure to find support for our tested hypothesis. The panels of Figure 1 suggest that for White participants, there is a significant difference in the CA relationship by Target Race when confidence is medium-low confidence but in no other confidence bin. To investigate this relationship further, inferential confidence intervals (ICI) comparing Target Race for each confidence bin were calculated (Table 1). ICIs indicated that accuracy was higher for White than Asian targets at all confidence levels except medium-high confidence.

The CA relationship was next examined by calculating the c-index, O/U, and ANDI (Brewer & Wells, 2006; Yaniv et al., 1991). These indices used suspect identifications; that is, correct identifications and the estimated innocent suspect identification rate (i.e., target-absent false identifications divided by resultant Tredoux’s *E*; Tredoux, 1998; A. M. Smith et al., 2020; Fitzgerald et al., 2023). Table 2 presents these indices. Inferential confidence intervals (ICIs; Tryon, 2001) were used for pairwise comparisons between White and Asian targets, but none of the differences were significant. Notably, calibration was strong. This is in line with the CAC curves, as the distance of each curve from the identity line was small and similar across Target Race for half of the confidence bins. In contrast to what was suggested by the CAC curves for some levels of confidence, participants did not differ overall in over- or underconfidence.

In summary, we expected a stronger CA relationship for same-race than cross-race faces. For our White participants, while the regression model and CAC curves indicated a difference in the CA relationship for White and Asian Targets identified by White participants, the calibration indices did not. Furthermore, as expected, CAC curves indicated that participants had greater overconfidence with cross-race compared to same-race faces, although O/U was not significantly different.

## 5. Discussion

This study is the first study, to the best of our knowledge, that investigated the CA relationship with White and Asian participants and faces (also see S. M. Smith et al., 2001), using sequential lineups (also see Wylie et al., 2015), and CAC curves. Given the importance of eyewitness confidence in jury decision-making and the fact that mistaken eyewitness testimony is a major cause of wrongful convictions (Garrett et al., 2020), our findings have significant implications. We discuss each of our key findings below.

We found a CRE for White participants, which affected their CA relationship–but not Asian participants. The lack of a CRE for Asian participants may be due to their contact with White individuals in their daily lives. Out of 203 Asian participants, 110 reported being originally from the USA, 187 reported currently residing in the USA, and 149 reported having lived longest in the USA. Given their experience in the USA and the fact that the predominant race in most parts of the USA is White, it is perhaps unsurprising that our Asian participants did not show a CRE. Other researchers have also found a CRE for the majority race but not for the numerical minority race (e.g., Chiroro & Valentine, 1995; Havard et al., 2017, 2025; Helm & Spearing, 2025; Stelter et al., 2023; Zhou et al., 2019). Indeed, the CRE is a small effect, declining over time, and predominantly observed with White participants (Lee & Penrod, 2022). Evidence for the CRE has also been inconclusive among racial groups that are numerical minorities in their countries of residence (for the CRE minority effect argument, see Töredi et al., 2024). The differing nationalities of our participants may have influenced their level of contact with faces from different racial groups, thereby affecting their CRE (likely more so among our Asian than White participants; see Töredi et al., 2024). Further empirical work would clarify whether this is the case. We also encourage future research to use more ecologically valid retention intervals (e.g., days after encoding) to improve generalizability. Indeed, retention interval has been shown to moderate the CRE (longer retention intervals are associated with a stronger CRE; Meissner & Brigham, 2001).

Confidence reliably predicted accuracy for both White and Asian participants. This finding is consistent with previous literature: confidence typically predicts accuracy under pristine lineup conditions (Wixted & Wells, 2017). Here, we found that, under pristine lineup conditions where there is a White and an Asian culprit, confidence is a reliable indicator of the eyewitness’s likely accuracy. This result is relevant to practice because confidence is the primary piece of information used to evaluate an eyewitness’s reliability and, therefore, the weight given to their testimony (Garrett et al., 2020, 2023). This study showed that the CA relationship can be reliable with White eyewitnesses’ East Asian identifications from sequential lineups under pristine lineup conditions.

Within the general CA relationship literature, relatively little attention has been given to cross-race conditions (cf. Dodson & Dobolyi, 2016; Grabman et al., 2019; Nguyen et al., 2017; S. M. Smith et al., 2001). Only one study to date considered the CA relationship for White and Asian participants and targets using a lineup paradigm (S. M. Smith et al., 2001), and one study to date considered the CA relationship for different race groups using sequential lineups (Wylie et al., 2015). This study is the first in the literature to find that White participants’ CA relationship differs for White and Asian targets using a sequential lineup. Confidence was expected to moderate the relationship between eyewitness accuracy and race, and the CA relationship was expected to differ for same-race and cross-race identifications. Because target race did not influence identification accuracy for Asian participants, our findings on whether race influenced the CA relationship were inconclusive. Therefore, the CA relationship findings were only forensically informative here for White participants. Different CA relationship indices suggested different conclusions about whether the CA relationship varies by race, in part because they use lineup decisions differently. All identifications were used in the regression models, whereas only suspect identifications (actual for target-present lineups, estimated for target-absent lineups) were used in calibration and CAC analyses. Regression and CAC analysis suggested a stronger CA relationship for same-race (cf. cross-race) targets while calibration indices suggested no difference in the CA relationship across target races.

The most forensically relevant analysis, inspection of the CAC curves, indicated a difference in the CA relationship as a function of race. For White participants, confidence was a better predictor of accuracy when the target was White than Asian for all levels of confidence except medium-high confidence (71–91%). Previous studies have found different CA relationships for different target races (Dodson & Dobolyi, 2016; Grabman et al., 2019; Wylie et al., 2015; cf. Nguyen et al., 2017). For instance, Dodson and Dobolyi found that White participants were better calibrated with same-race targets than cross-race (Black) targets, even though there were only small differences in calibration based on the overconfidence observed with cross-race targets. Dodson, Dobolyi, and Grabman et al. looked at calibration curves (cf. CAC curves) but did not inferentially compare performance at each confidence category, and Wylie et al. used correlations. We drew similar conclusions to previous findings, but using CAC curves, regressions, and calibration indices. The CAC curves indicated the differing CA relationship derived from medium-low confidence identifications; participants were overconfident with Asian targets and underconfident with White targets. This finding aligns with prior research demonstrating more overconfidence in cross-race identifications (Dodson & Dobolyi, 2016). The reason this was not observed in the calibration indices over-under analysis may be that it collapses across confidence bins to look at the overall CA relationship.

Finally, one difference between our findings and those of Dodson and Dobolyi (2016) is that they observed small differences in the CA relationship for same- and cross-race targets across all confidence categories, whereas we found differences in three out of the four confidence bins. We found that participants who had low, medium-low, or high confidence in their identifications (but not medium-high) were better calibrated for White targets than Asian targets. Participants who had medium-high confidence (i.e., 71–90%) in their identifications were well-calibrated and showed a similar CA relationship across White and Asian targets, such that they were similarly underconfident. Grabman et al. (2019) reported a similar CA relationship for White and Black targets when participants had high confidence. Inspection of their Figure 3c shows that the 95% confidence intervals for same-race and cross-race identifications overlapped from approximately 60% confidence onwards. It is clear from their figure that in the highest confidence category, the difference was not significant, whereas we found a difference when confidence was 91% and above. This is important as high-confidence is highly persuasive to legal decision-makers (Garrett et al., 2020).

### Theoretical Perspectives

It is important to consider these findings in relation to the theoretical accounts of the CA relationship. There are two main theoretical accounts of the confidence-accuracy relationship: the optimality hypothesis (Deffenbacher, 1980) and the constant likelihood ratio model (Semmler et al., 2018).

The optimality hypothesis (Deffenbacher, 1980) suggests that optimal encoding, storage, and retrieval conditions influence an eyewitness’s ability to accurately discriminate a guilty suspect from an innocent suspect, their confidence level, and the CA relationship. For example, eyewitnesses who view a culprit for longer are expected to be both more accurate and more confident, and to have a stronger CA relationship than eyewitnesses who view a culprit for a shorter period of time. Non-optimal conditions are proposed to have a negative effect on the CA relationship because they do not allow participants to adequately reflect on their accuracy. Several studies have supported this hypothesis (e.g., Giacona et al., 2021; Lockamyeir et al., 2020; Sauer et al., 2019).

The constant likelihood ratio model (Semmler et al., 2018) takes a different view, leading to quite different predictions than the optimality hypothesis (Deffenbacher, 1980). The constant likelihood ratio model posits that estimator variables should not influence the CA relationship because eyewitnesses will adjust their confidence level based on their encoding conditions. Accordingly, the model proposes that confidence will be a reliable reflector of identification accuracy regardless of how optimal encoding conditions are. In support of the constant likelihood ratio model, several studies have found that the CA relationship is maintained despite sub-optimal encoding conditions (e.g., Carlson et al., 2023; Flowe et al., 2017; Harvey et al., 2020; Nguyen et al., 2017; Nyman et al., 2019; Semmler et al., 2018).

If a cross-race scenario is suboptimal for encoding (e.g., lack of perceptual expertise) or retrieval (e.g., lack of motivation)—as suggested by the existence of the CRE (Meissner & Brigham, 2001)—then the optimality hypothesis (Deffenbacher, 1980) predicts the CA relationship to be stronger for same-race compared to cross-race lineups. In contrast, the constant likelihood ratio model (Semmler et al., 2018) predicts no difference in the CA relationship based on target race because eyewitnesses will adjust their confidence based on their actual recognition ability. Our findings support the predictions of the optimality hypothesis for White eyewitnesses because our CAC curves suggested that, for three out of four confidence bins we employed, participants had a stronger CA relationship for same-race than cross-race targets. However, not observing this for all confidence bins may suggest that individuals adjust their confidence based on their recognition ability for some confidence levels, as the constant likelihood ratio model suggests. But alternatively, the lack of difference may simply reflect a Type 2 error.

## 6. Conclusions

In conclusion, we found that White eyewitnesses show a weaker CA relationship for East Asians than White culprits, similar to prior studies. We found this using sequential lineups and various calibration indices, which were rarely used in previous studies. Our findings are consistent with the optimality hypothesis and indicate a practically relevant boundary condition for the CA relationship. Taken together, our findings indicate that target race influences the reliability of confidence as an index of eyewitness accuracy. However, additional research is needed before drawing strong conclusions or recommending cautionary changes to how legal decision-makers evaluate eyewitness confidence. In particular, replication across a broader range of racial pairings, lineup procedures, fairer lineups, and applied settings is necessary to determine the robustness and practical boundaries of these effects. Until such evidence is available, the present results should be viewed as highlighting a potential source of variability in the confidence–accuracy relationship, that is, a potential boundary condition.

## Figures and Tables

**Table 1:** 95% Inferential Confidence Intervals Comparing Target Race for Each Confidence Bin Based on Confidence Accuracy Characteristics.

	Target Race	
Confidence Bin	White	Asian
Low	[0.76, 0.83]	[0.58, 0.69]
Medium-low	[0.80, 0.89]	[0.55, 0.68]
Medium-high	[0.88, 0.93]	[0.80, 0.90]
High	[0.95, 0.99]	[0.82, 0.93]

**Table 2:** Calibration Indices and 95% Inferential Confidence Intervals Comparing Target Races.

	Target Race	
Calibration Indices	White	Asian
c-index	0.03 [−0.009, 0.07]	0.01 [−0.005, 0.04]
O/U	−0.11 [−0.21, −0.01]	−0.04 [−0.13, 0.04]
ANDI	0.07 [0.01, 0.13]	0.07 [0.02, 0.13]

## Data Availability

This study was preregistered (osf.io/9jnzc: https://doi.org/10.17605/OSF.IO/J9NZC) on the Open Science Framework (OSF), including the hypotheses, method, sample size, exclusion criteria, and analytic plan. The raw data (stripped of any personally identifying information) and analysis scripts are shared publicly via the OSF (osf.io/9abs: https://osf.io/j9nzc/overview#:~:text=https%3A//osf.io/nx5mf, accessed on 20 February 2021).

## Notes

1 Other Asian countries reported by participants to be as country of origin are: *n* = 11 India, *n* = 9 China, *n* = 9 Korea, *n* = 5 Vietnam, *n* = 5 the Philippines, *n* = 4 Hong Kong, *n* = 3 Indonesia, *n* = 3 Pakistan, *n* = 2 Taiwan, *n* = 2 Malaysia, *n* = 1 Bangladesh, *n* = 1 Kazakhstan, *n* = 1 Liberia, *n* = 1 Nepal, *n* = 1 Singapore, *n* = 1 Thailand, *n* = 1 Laos, and *n* = 1 Trinidad.

2 *n* = 11 India, *n* = 9 China, *n* = 2 Hong Kong, *n* = 2 Malaysia, *n* = 2 Pakistan, *n* = 1 Kazakhstan, *n* = 1 Korea, *n* = 1 Singapore, *n* = 1 Taiwan, *n* = 1 Thailand, *n* = 1 Vietnam, *n* = 1 Pakistan.

3 *n* = 6 Poland, *n* = 4 Italy, *n* = 2 Hungary, *n* = 2 Ireland, *n* = 1 Egypt, *n* = 1 Finland, *n* = 1 Greece, *n* = 1 Latvia, *n* = 1 Lithuania, *n* = 1 Norway, *n* = 1 Romania, *n* = 1 South Africa

4 *n* = 2 Hungary, *n* = 2 Ireland, *n* = 2 Italy, *n* = 1 Egypt, *n* = 1 Finland, *n* = 1 France, *n* = 1 Greece, *n* = 1 Norway, *n* = 1 Poland, *n* = 1 Romania, *n* = 1 South Africa, *n* = 1 Netherlands, *n* = 1 United Arab Emirates

5 TM and © 2008 Entertainment Rights Distribution Limited. All rights reserved.

## Supplementary Materials

The following supporting information can be downloaded at: https://www.mdpi.com/article/10.3390/bs16020257/s1.
