# Supplementary material for: Does Eyewitness Confidence Calibration Vary by Target Race?
Source: Behav Sci (Basel). 2026 Feb 10;16(2):257. doi: 10.3390/bs16020257 (PMC12938357; doi:10.3390/bs16020257)
Supplement: Supplementary file 1 [file behavsci-16-00257-s001.zip › behavsci-4080883-supplementary.pdf]

Supplementary Materials

Table S1

Lineup Decisions for each Lineup (percentages)

	Correct ID	False ID from TP	Incorrect rejections	Correct Rejections	False ID from TA
			White participants		
W Lineup 1	80.20	11.45	8.33	38.67	61.32
W Lineup 2	92.45	4.71	2.83	82.10	17.89
A Lineup 1	57.54	33.01	9.43	30.52	69.47
A Lineup 2	86.45	10.41	3.12	40.56	59.49
			Asian participants		
W Lineup 1	72.30	15.38	12.30	51.21	48.78
W Lineup 2	91.25	5	3.75	68.49	31.50
A Lineup 1	56.41	33.33	10.25	41.66	58.33
A Lineup 2	89.55	4.47	5.97	43.37	56.62

Table S2

Resultant Tredoux's E for each Lineup

	Target-present lineups		Target-absent lineups	
	White Participants	Asian Participants	White Participants	Asian Participants
White Lineup 1	1.30 [1.12, 1.53]	1.44 [1.18, 1.84]	4.11 [3.26, 5.56]	3.03 [2.18, 4.94]
White Lineup 2	1.10 [1.01, 1.20]	1.11 [1.00, 1.23]	3.48 [2.44, 6.02]	4.16 [3.43, 5,29]
Asian Lineup 1	2.14 [1.79, 2.68]	2.14 [1.75, 2.74]	1.96 [1.54, 2.69]	1.55 [1.23, 2.09]
Asian Lineup 2	1.24 [1.10, 1.45]	1.10 [0.99, 1.23]	2.49 [1.95, 3.45]	2.36 [1.83, 3.33]

Note: Intervals in square brackets are 95% confidence intervals.

Table S3

Lineup Member Selections by Target (percentages of identifications)

	Target-present lineups						Target-absent lineups					
	1	2	3	4	5	6	1	2	3	4	5	6
						White participants						
W Lineup 1	2.08	**80.20**	2.08	2.08	2.08	3.12	16.66	39.39	13.63	13.63	3.03	12.12
W Lineup 2	1.07	0	**93**	2	1.07	1.07	11.7	5.88	11.76	41.17	0	29.41
A Lineup 1	4.16	23.95	3.12	2.08	**63.54**	3.12	69.69	4.54	3.03	6.06	12.12	4.54
A Lineup 2	0	0	5.37	**89.27**	5.37	0	19.04	1.58	4.76	58.73	3.17	12.69

Note: Targets are bolded.

Confidence and the Confidence-Accuracy Relationship of Asian Participants

Confidence was expected to be a reflector of accuracy for both participants. To examine this, confidence was regressed to predict identification accuracy in a model that also included participant as a random effect. Confidence predicted identification accuracy for Asian participants, $z = 6.55$, $p < .001$. Next, whether confidence interacted with the Target Race to predict lineup accuracy was examined—again using a multilevel binomial logistic regression model, but now including Target Race, confidence, and their interaction as fixed effects as well as participant as a random effect. It was hypothesised that confidence would interact with Target Race. However, this interaction was not observed for Asian participants, $z = 0.72$, $p = .47$, indicating a similar CA relationship for same-race and cross-race targets.

The CAC curves for Target Race indicated a positive CA relationship for both target races (see Figure S1). For the low confidence identifications, Asian participants were underconfident with White targets but overconfident with Asian targets. Medium-low confidence identifications were well-calibrated for Asian targets but not for White targets for which they were underconfident. Furthermore, Asian participants were well-calibrated for both White targets and Asian targets when they had medium-high confidence or high confidence in their identifications. For Asian participants, there were significant differences in CA for low and medium-low confidence bins (confidence intervals do not overlap), but there were no significant differences in CA for high and medium-high confidence bins (standard errors overlap). To investigate this relationship further, inferential confidence intervals (ICI) comparing target race for each confidence bin were calculated for each Participant Race (see Table S4). The ICIs suggested that the CA relationship significantly differed for Asian participants for low and medium-low confidence bins but not for medium-high and high confidence bins. These findings are in line with CAC curve suggestions.

Figure S1

CAC Curves by Target Race, using Standard Error Bars and 95% Confidence Intervals

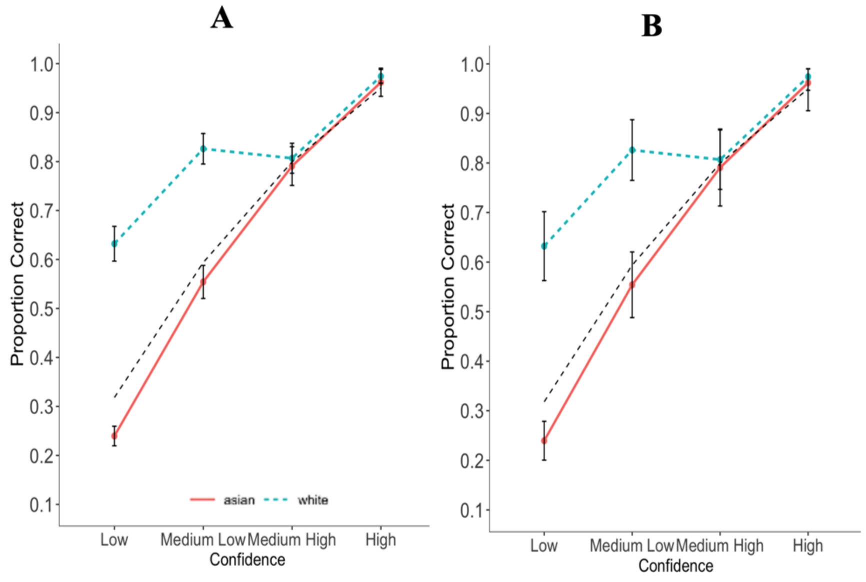

Note. Panel A plots standard error bars while B plots 95% confidence intervals.

Table S4

95% Inferential Confidence Intervals Comparing Target Race for Each Confidence Bin Based on Confidence Accuracy Characteristics

	Participant Race	
Confidence Category	White Participants	Asian Participants
Low	WT: [.76, .83]	WT: [.72, .83]
	AT: [.58, .69]	AT: [.42, .48]
Medium- low	WT: [.80, .89]	WT: [.82, .91]
	AT: [.55, .68]	AT: [.59, .69]
Medium-high	WT: [.88, .93]	WT: [.81, .90]
	AT: [.80, .90]	AT: [.75, .87]
High	WT: [.95, .99]	WT: [.94, .99]
	AT: [.82, .93]	AT: [.92, .99]

The CA relationship was next examined by calculating calibration indices. Specifically, the calibration index (*c-index*), the over/underconfidence (*OU*), and the adjusted normalised discrimination index (*ANDI*; Yaniv et al., 1991) were calculated. Table S5 below presents the results of calibration analyses. Inferential confidence intervals (ICIs; Tryon, 2001) were used for pairwise comparisons between White and Asian targets, but none of the differences were significant. Notably, calibration was strong. This is in line with the CAC curves, as the distance of each curve to the identity line appears relatively similar for at least two of the confidence bins. There were no differences based on Target Race on how well confidence discriminates between guilty and innocent suspects. In contrast to what was suggested by the CAC curves for some levels of confidence, participants did not differ overall in over- or underconfidence.

Table S5

Calibration Indices and 95% Inferential Confidence Intervals Comparing Target Races

	Participant Race	
Calibration Indices	Asian participants	White participants
c-index	WT: c = .02 [-.01, .05]<br>AT: c = .001 [-.005, .003]	WT: c = .03 [-.009, .07]<br>AT: c = .01 [-.005, .04]
OU	WT: *OU* = -.09 [-.19, .01]<br>AT: *OU* = .01 [-.01, .04]	WT.: *OU* = -.11 [-.21, -.01]<br>AT: *OU* = -.04 [-.13, .04]
ANDI	WT: *ANDI* = .11 [.01, .21]<br>AT: *ANDI* = .26 [.06, .47]	WT: *ANDI* = .07 [.01, .13]<br>AT: *ANDI* = .07 [.02, .13]

In summary, for Asian participants, regression models and calibration indices suggested a similar CA relationship for Target Race while CAC curves and inferential confidence intervals outline that it was not the case for low and medium-low confidence bins.

Confidence-Accuracy Relationship: Full Set of Lineups

This section reports the full results when the biased lineup was not excluded from the analyses, as per our preregistration.

It was hypothesised that confidence would interact with Target Race irrespective of Participant Race. Consistent with this hypothesis, the Target Race and confidence interaction was significant for White participants, $z = 2.46$, $p = .01$, indicating a stronger confidence-accuracy (CA) relationship for same-race than cross-race targets. However, this interaction was not observed for Asian participants, $z = 0.72$, $p = .47$, indicating a similar CA relationship for same-race and cross-race targets.

CAC curves (Mickes, 2015) and calibration analyses (Brewer & Wells, 2006) were used to examine the CA relationship for same-race versus cross-race decisions. First, CAC curves were plotted. Since this study did not use a designated innocent suspect, the innocent suspect identification rate was calculated by dividing the target-absent filler identification rate by the lineup's resultant functional size (as measured by Tredoux's *E*; Smith et al., 2020a; Fitzgerald, 2020). Confidence was categorised as low (0-50 %), medium-low (51-70 %), medium-high (71-90 %), or high (91-100 %). Identity lines were determined by calculating the weighted mean accuracy for n each confidence bin. In Figure 2, standard error bars and 95% confidence interval bars were employed as separate panels and separately for Participant Race. Both representations were utilised to provide comprehensive insights into potential differences between confidence bins across Target Race conditions.

The CAC curves for Target Race and Participant Race indicated a positive CA relationship in all four cases (see Figure S2). The CAC curves were similar for White and Asian participants for all confidence bins. For the low confidence identifications, White and Asian participants were underconfident with both White and Asian targets. Across Participant

Race groups, medium-low confidence identifications were well-calibrated for Asian targets but not for White targets for which they were underconfident. Furthermore, for Asian participants, medium-high confidence identifications were well-calibrated for White targets but not for Asian targets for which they were overconfident. High confidence identifications for Asian participants and both medium-high and high confidence identifications for White participants were underconfident with White targets and overconfident with Asian targets.

Figure S2

CAC Curves for Participant Race by Target Race, using Standard Error Bars and 95% Confidence Intervals

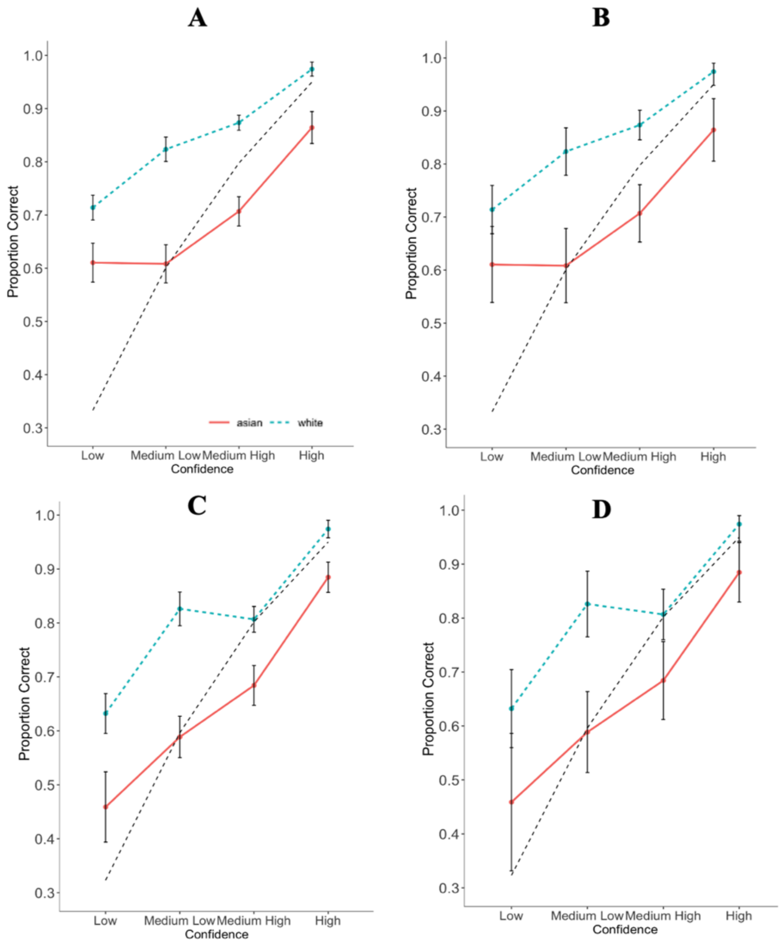

Note. Panels A and C plot standard error bars, B and D plot 95% confidence intervals. The top panels represent White participants, and the bottom panels represent Asian participants.

The panels of Figure 2 suggest that, for both White and Asian participants, there were significant differences in CA relationships across the Target Race in three confidence bins: medium-low, medium-high and high (confidence intervals do not overlap), whereas figures were not informative about the low confidence bin. To investigate this relationship further, inferential confidence intervals around CAC confidence bins were calculated for each Participant Race (see Table S6). The inferential confidence intervals suggested that the CA relationship significantly differed for White participants for all confidence bins in a way that it was stronger for same-race than cross-race targets. The inferential confidence intervals suggested that the CA relationship significantly differed for Asian participants for all confidence bins—except the high confidence bin.

Table S6

Inferential Confidence Intervals for Each Confidence Bin of Confidence Accuracy Characteristic Curves for Participant Race

	Participant Race	
Confidence Category	White Participants	Asian Participants
Low	WT: [.76, .83]	WT: [.71, .83]
	AT: [.58, .69]	AT: [.44, .64]
Medium- low	WT: [.81, .88]	WT: [.82, .91]
	AT: [.58, .69]	AT: [.55, .67]
Medium-high	WT: [.88, .93]	WT: [.82, .89]
	AT: [.68, .77]	AT: [.62, .74]
High	WT: [.95, .99]	WT: [.94, .99]
	AT: [.79, .88]	AT: [.85, .94]

Note. WT = White target, AT = Asian target.

The confidence-accuracy relationship was next examined by calculating calibration indices. Specifically, the calibration index (*c-index*), the over/underconfidence (*OU*), and the adjusted normalised discrimination index (*ANDI*; Yaniv et al., 1991) were calculated. Table S7 below presents the results of calibration analyses. Inferential confidence intervals (ICIs; Tryon, 2001) were used for pairwise comparisons between White and Asian targets within

each Participant Race group, but none of the differences were significant. Notably, calibration was strong regardless of Participant Race. This is in line with the CAC curves, as the distance of each curve to the identity line appears relatively similar. There were no differences based on Target Race on how well confidence discriminates between guilty and innocent suspects. In contrast to what was suggested by the CAC curves for some levels of confidence, participants did not differ overall in over- or underconfidence.

Table S7

Calibration Indices and 95% Inferential Confidence Intervals for Target Race Comparisons

	Participant Race	
Calibration Indices	Asian participants	White participants
c-index	WT: *c* = .02 [-.01, .05]<br>AT: *c* = .01 [.002, .01]	WT: *c* = .03 [-.009, .07]<br>AT: *c* = .02 [.001, .04]
OU	WT: *OU* = -.09 [-.18, .001]<br>AT: *OU* = .03 [-.02, .09]	WT.: *OU* = -.11 [-.21, -.01]<br>AT: *OU* = -.01 [-.11, .09]
ANDI	WT: *ANDI* = .11 [.02, .20]<br>AT: *ANDI* = .07 [.01, .17]	WT: *ANDI* = .07 [.01, .13]<br>AT: *ANDI* = .04 [.001, .08]

Note. WT = White target, AT = Asian target.

In summary, for White participants, regression and CAC curves indicated that confidence was a better overall predictor of accuracy for same-race than cross-race faces. In contrast, the calibration indices indicated that there were no differences in calibration. For Asian participants, CAC curves indicated that confidence was a better overall predictor of accuracy for cross-race than same-race faces. However, regression and the calibration indices indicated that there were no differences in calibration.
